# The landscape of *N*^1^-methyladenosine (m^1^A) modification in mRNA of the decidua in severe preeclampsia

**DOI:** 10.17305/bb.2024.10532

**Published:** 2024-12-01

**Authors:** Jing Tong, Hua Li, Liang Zhang, Cong Zhang

**Affiliations:** 1 Department of Reproductive Medicine, Ren Ji Hospital, Shanghai Jiao Tong University School of Medicine, Shanghai, China; 2 Shanghai Key Laboratory for Assisted Reproduction and Reproductive Genetics, Shanghai, China; 3 Jinan Maternal and Child Health Care Hospital Affiliated to Shandong First Medical University, Jinan, Shandong, China; 4 Research Center of Translational Medicine, Jinan Central Hospital Affiliated to Shandong First Medical University, Jinan, Shandong, China; 5 Shandong Provincial Key Laboratory of Animal Resistance Biology, College of Life Sciences, Shandong Normal University, Jinan, Shandong, China; 6 Shandong Provincial Key Laboratory of Reproductive Medicine, Jinan, Shandong, China

**Keywords:** Decidua, decidualization, *N*^1^-methyladenosine (m^1^A), methylated RNA immunoprecipitation sequencing (MeRIP-seq), preeclampsia (PE), vascular dysfunction

## Abstract

Recent discoveries in mRNA modification have highlighted *N*^1^-methyladenosine (m^1^A), but its role in preeclampsia (PE) pathogenesis remains unclear. In this study, we utilized methylated RNA immunoprecipitation sequencing (MeRIP-seq) and RNA sequencing (RNA-seq) to identify m^1^A peaks and the expression profile of mRNA in the decidua of humans with early-onset PE (EPE), late-onset PE (LPE), and normal pregnancy (NP). We assessed the m^1^A modification patterns in preeclamptic decidua using 10 m^1^A modulators. Our bioinformatic analysis focused on differentially methylated mRNAs (DMGs) and differentially expressed mRNAs (DEGs) in pairwise comparisons of EPE vs NP, LPE vs NP, and EPE vs LPE, as well as m^1^A-related DEGs. The comparisons of EPE vs NP, LPE vs NP, and EPE vs LPE identified 3110, 2801, and 2818 DMGs, respectively. We discerned three different m^1^A modification patterns from this data. Further analysis revealed that key PE-related DMGs and m^1^A-related DEGs predominantly influence signaling pathways critical for decidualization, including cAMP, MAPK, PI3K-Akt, Notch, and TGF-β pathways. Additionally, these modifications impact pathways related to vascular smooth muscle contraction, estrogen signaling, and relaxin signaling, contributing to vascular dysfunction. Our findings demonstrate that preeclamptic decidua exhibits unique mRNA m^1^A modification patterns and gene expression profiles that significantly alter signaling pathways essential for both decidualization and vascular dysfunction. These differences in m^1^A modification patterns provide valuable insights into the molecular mechanisms influencing the decidualization process and vascular function in the pathogenesis of PE. These m^1^A modification regulators could potentially serve as potent biomarkers or therapeutic targets for PE, warranting further investigation.

## Introduction

Preeclampsia (PE) is a multisystem syndrome that uniquely occurs during human pregnancy. It is characterized by the new onset of hypertension, proteinuria, and other signs of maternal vascular damage after 20 weeks of gestation. PE affects approximately 8% of first-time pregnancies worldwide each year, impacting eight million mother-infant pairs. It is recognized as one of the leading causes of maternal and perinatal morbidity and mortality [1, 2]. Severe PE (sPE) is diagnosed based on a further elevation of systolic pressure ≥ 160 mm Hg or diastolic pressure ≥ 110 mm Hg, or any of the following: thrombocytopenia, impaired liver function, progressive renal insufficiency, pulmonary edema, and the new onset of cerebral or visual disturbances [3].

Currently, the etiology of PE remains unclear, but dysregulated decidualization has been a subject of interest in studying the genesis of PE. Previous studies have confirmed and augmented the results through global transcriptional profiling of decidua in sPE [4]. It was surprising to find that the decidual gene transcription profile was altered in sPE. Additionally, decidual cells from patients with sPE, when dedifferentiated in vitro, failed to redecidualize in culture [5]. Therefore, the decidua is considered a prime candidate for the genesis of PE [6]. In humans, decidualization, the formation of the decidua, is independent of the presence of a conceptus. It is a progressive process that involves hormonally regulated differentiation of human endometrial stromal cells (ESCs), morphologically transforming into enlarged round-shaped cells from a fibroblast-like population driven by genetic reprogramming [7].

*N*^1^-methyladenosine (m^1^A) is a recently identified mRNA modification that is found enriched around the first codon upstream of the first splice site and alternative translation initiation sites. This modification is highly conserved in human cells and is responsive to physiological conditions. It correlates positively with protein production, indicating a functional role in promoting translation of methylated mRNA [8]. m^1^A modification is modified by the m^1^A methyltransferases, or “writers”, such as *TRMT10C*, *TRMT61B*, *TRMT6,* and *TRMT61A*, and removed by the demethylases, or “erasers” including *ALKBH1* and *ALKBH3*. It is recognized by m^1^A-binding proteins *YTHDF1*, *YTHDF2*, *YTHDF3*, and *YTHDC1*, also known as “readers” [9, 10]. These ten regulatory genes play an essential role in the process of modifying m^1^A. However, little is known about the precise location and biogenesis of m^1^A methylation in mRNA of decidual tissue in sPE.

To reveal mechanisms in decidualization and explore potential epigenetic biomarkers in sPE, this study aimed to investigate the m^1^A methylation atlas in mRNA in decidual tissue. Two strategies were employed: (i) an m^1^A-specific and in-depth bioinformatics analysis of m^1^A in mRNA obtained from individuals with sPE and normal pregnancies (NPs) using methylated RNA immunoprecipitation sequencing (MeRIP-seq) and (ii) an analysis of m^1^A modification patterns by RNA sequencing (RNA-seq) in decidual tissue of sPE and NPs.

## Materials and methods

### Participants and samples

Nine decidua samples were collected from patients with an NP (*n* ═ 3), EPE (*n* ═ 3), and LPE (*n* ═ 3) at the Department of Obstetrics and Gynecology, Ren Ji Hospital, Shanghai Jiao Tong University School of Medicine, between January 2018 and July 2019. EPE was defined as the onset of symptoms before 34 weeks of gestation, while LPE was defined as the onset of symptoms at or after 34 weeks of gestation [3]. Patients with multiple pregnancies and other complications, such as maternal diabetes, thyroid dysfunction, and abnormal placental structure were excluded. Human decidua basalis was collected from the placental bed during caesarean section, separated into centrifuge tubes, and snap-frozen in liquid nitrogen for further analysis. 

### MeRIP-seq and RNA-seq

MeRIP-Seq was performed as described in the published procedure with slight modifications [11]. Total RNA was isolated using Trizol Reagent (Life Technologies) and then quantified using the NanoDrop ND-1000 instrument (Thermo Fisher Scientific, MA, USA). The RNA purity was assessed by ensuring that its OD260/OD280 value fell between 1.8 and 2.1. Next, the total RNA was depleted of rRNA using Ribo-zero (Illumina) and fragmented using an RNA fragmentation reagent (Thermo Fisher Scientific). All of the RNA fragments were subjected to immunoprecipitation using the GenSeqTM m^1^A-MeRIP Kit (GenSeq Inc., China), following the manufacturer’s instructions. The input samples, which underwent no immunoprecipitation, were used to generate RNA-seq libraries. Both the input and immunoprecipitation samples were used to construct libraries using the NEBNext^®^ Ultra II Directional RNA Library Prep Kit (New England Biolabs, Inc., USA). The libraries were then sequenced on a HiSeq platform (Illumina).

### Sequencing data analysis

Paired-end reads were obtained from the Illumina HiSeq 4000 sequencer and underwent quality control using Q30. Clean reads were then generated after trimming the 3’ adaptors and removing low-quality reads using the Cutadapt software (v1.9.3). These clean reads from all libraries were aligned to the UCSC HG19 reference genome using the Hisat2 software. For the m^1^A IP-seq data, the MACS software was utilized to identify methylated sites on RNAs (peaks) [12]. Differential methylated sites were determined using the DiffReps software [13]. Custom scripts were developed to select the peaks that overlapped with mRNA exons [14]. The mRNAs that overlapped with these differentially methylated peaks and had a fold change (FC) of at least 2.0 (Log (FC) ≥ 1.0) and a *P* value less than or equal to 0.0001 were classified as differentially methylated mRNAs (DMGs). For the input-seq data, raw counts were obtained using the HTSeq software (v0.9.1) and normalized using EdgeR [15]. The differentially expressed mRNAs (DEGs) were defined as those with an FC of at least 2.0 (Log (FC) ≥ 1.0) and a *P* value less than or equal to 0.01.

### Bioinformatics analysis

Consensus clustering analysis was performed based on the expression of 10 m^1^A regulators using the Consensus Cluster Plus R package. We employed the consensus cumulative distribution function (CDF) and the delta area plot to evaluate cluster stability and robustness. Based on these criteria, we found that a value of *K* ═ 3 yielded the most stable and interpretable clustering solution. Consequently, the PE group was divided into three distinct clusters. Gene set variation analysis (GSVA) was then performed in the PE and NP groups using the GSVA package in R package. The gene sets for GSVA were obtained from the MSigDB database, specifically the “C2.cp. kegg. V 7.5.1. symbols” gene sets (accessed on 8 May 2022). The m^1^A-related DEGs were defined as the union of genes selected in m^1^A modification pattern pairwise comparisons, meeting the criteria of an FC of ≥2.0 (Log(FC) ≥1.0) with adjusted *P* values of <0.05. UpSetR was used for data exploration and generating set visualizations [16]. The DMGs, DEGs, and m^1^A-related DEGs were then subjected to functional and pathway enrichment analysis using the Gene Ontology (GO) and Kyoto Encyclopedia of Genes and Genomes (KEGG) databases. GO, the world’s largest source of information on the functions of genes (www.geneontology.org), and KEGG (www.genome.jp/kegg) provide insights into the high-level functions and utilities of biological systems, respectively.

### External validation of the mRNA expression of key m^1^A-related DEGs and m^1^A modulators

We selected a validation set consisting of gene expression data from 60 preeclamptic patients and 65 normal controls sourced from the Gene Expression Omnibus (GEO) dataset, specifically GSE60438. To normalize the gene expression data, we employed the “Scale” function, which facilitated subsequent comparative analyses.

**Figure 1.**
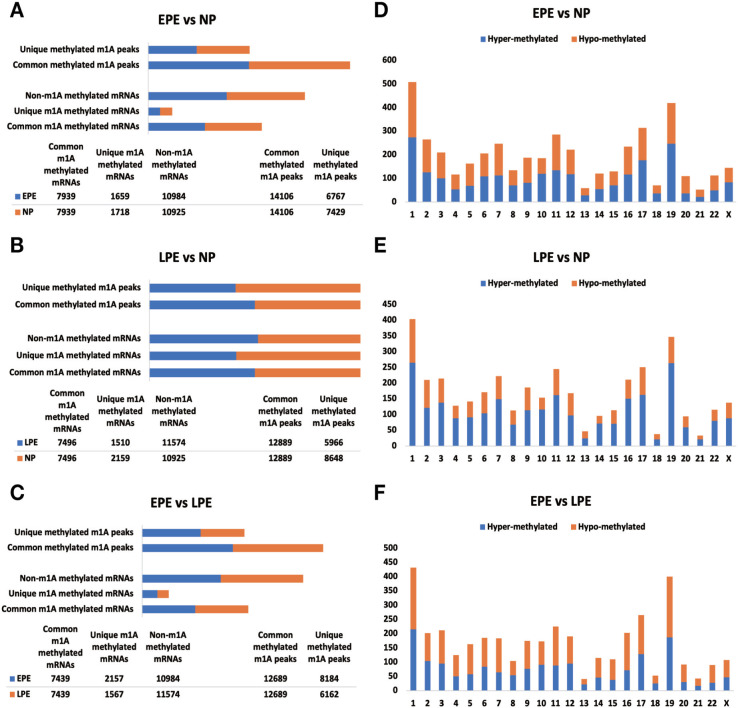
**The overview of the m^1^A methylation map in pairwise comparisons.** (A–C) The total number of unique and common methylated m^1^A peaks in EPE vs NP (A, upper section), LPE vs NP (B, upper section), and EPE vs LPE (C, upper section) comparisons; while the lower sections show the total number of unique, common, and non-m^1^A methylated mRNAs in EPE vs NP (A), LPE vs NP (B), and EPE vs LPE (C) comparisons; (D–F) The distribution of differentially methylated m^1^A sites with significance across chromosomes for three comparisons. From up to down are EPE vs NP (D), LPE vs NP (E), and EPE vs LPE (F). EPE: Early-onset preeclampsia; LPE: Late-onset preeclampsia; NP: Normal pregnancy.

### Ethical statement

The study was approved by the Ren Ji Hospital Research and Ethics Committee. All participants provided informed consent before the caesarean section.

### Statistical analysis

Statistical analyses and visualizations were conducted using R software (version 3.5.3, https://www.r-project.org/) and GraphPad Prism v6.0 for Mac (GraphPad; San Diego, CA, USA). The Student’s *t*-test was employed to assess the statistical differences between the two groups. For all analyses, a two-sided *P* value was utilized, with a significance level set at *P* < 0.05.

## Results

### Overview of the m^1^A methylation map in pairwise comparisons

The pairwise comparison of m^1^A peaks and m^1^A methylated mRNAs in the three groups, EPE vs NP, LPE vs NP, and EPE vs LPE, revealed unique m^1^A peaks and unique m^1^A methylated mRNAs in each comparison. In the EPE group, a total of 6767 unique m^1^A peaks and 1659 unique m^1^A methylated mRNAs were identified. Similarly, in the NP group, there were 7429 unique m^1^A peaks and 1718 unique m^1^A methylated mRNAs in the EPE vs NP comparison (Figure 1A). In the LPE group, there were 5966 unique m^1^A peaks and 1510 unique m^1^A methylated mRNAs; and 8648 unique m^1^A peaks and 2159 unique m^1^A methylated mRNAs in the NP group in the LPE vs NP comparison (Figure 1B). Further, a total of 8184 unique m^1^A peaks and 2157 unique m^1^A methylated mRNAs were obtained in the EPE group; and 6162 unique m^1^A peaks and 1567 unique m^1^A methylated mRNAs in the LPE group for the EPE vs LPE comparison (Figure 1C). Subsequently, all the differentiated methylated m^1^A peaks in each comparison were mapped to human chromosomes. They were distributed across all chromosomes, with higher abundance observed in chr1, chr17, and chr19, while no m^1^A peaks were found in chrY (Figure 1D–1F).

### GO and KEGG pathway analysis of DMGs

**Figure S1.**
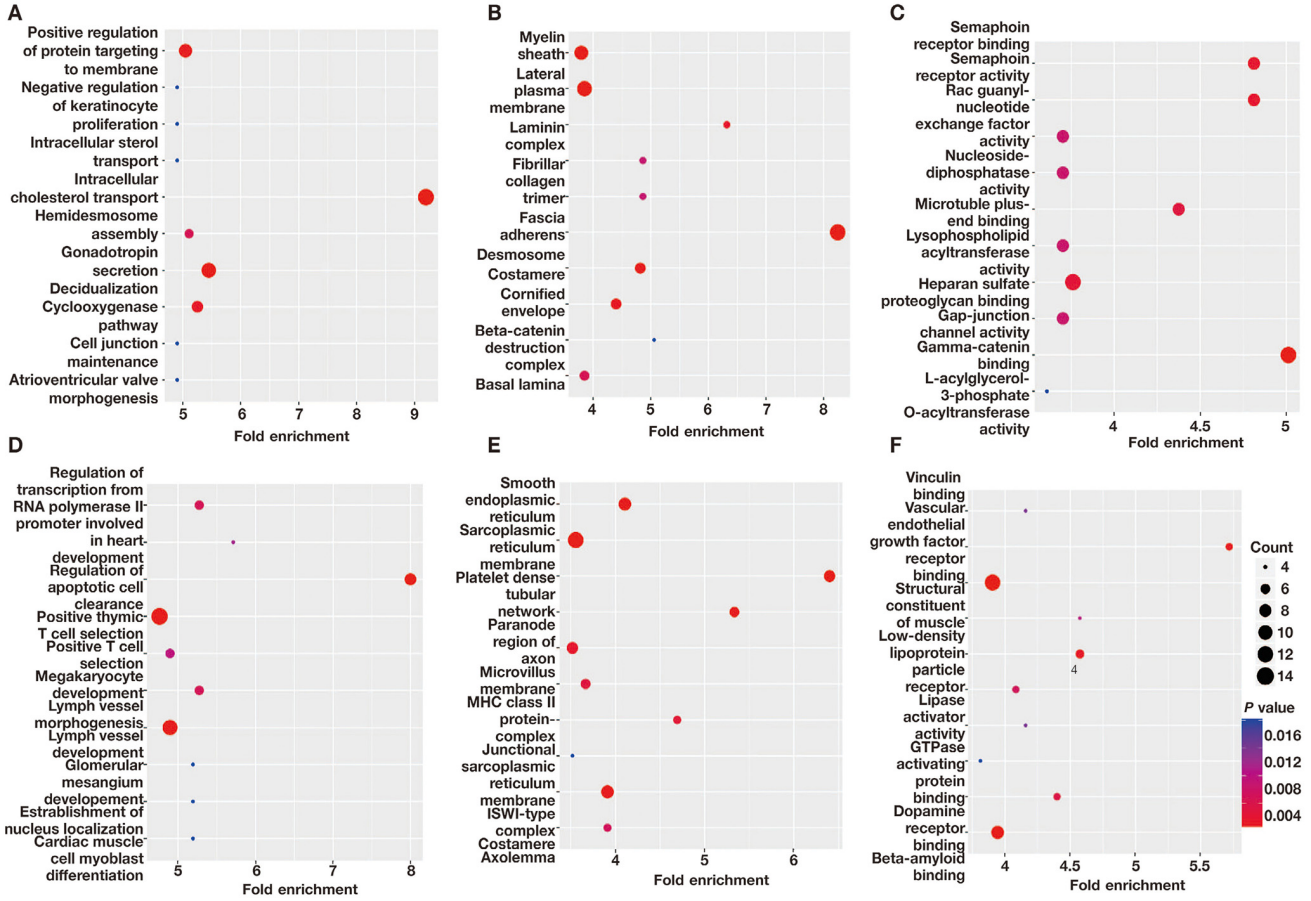
**The GO analysis of DMGs in the EPE vs NP comparison.** (A) The top ten significantly enriched BP terms among hypermethylated DMGs; (B) The top ten significantly enriched CC terms of hypermethylated DMGs; (C) The top ten significantly enriched molecular function (MF) terms of hypermethylated DMGs; (D) The top 10 significantly enriched biological process (BP) terms of hypomethylated DMGs; (E) The top 10 significantly enriched cellular component (CC) terms of hypomethylated DMGs; (F) The top 10 significantly enriched MF terms of hypomethylated DMGs. DMGs: Differentially methylated mRNAs; EPE: Early-onset preeclampsia; NP: Normal pregnancy; GO: Gene ontology; BP: Biological process; CC: Cellular component; MF: Molecular function.

**Figure S2.**
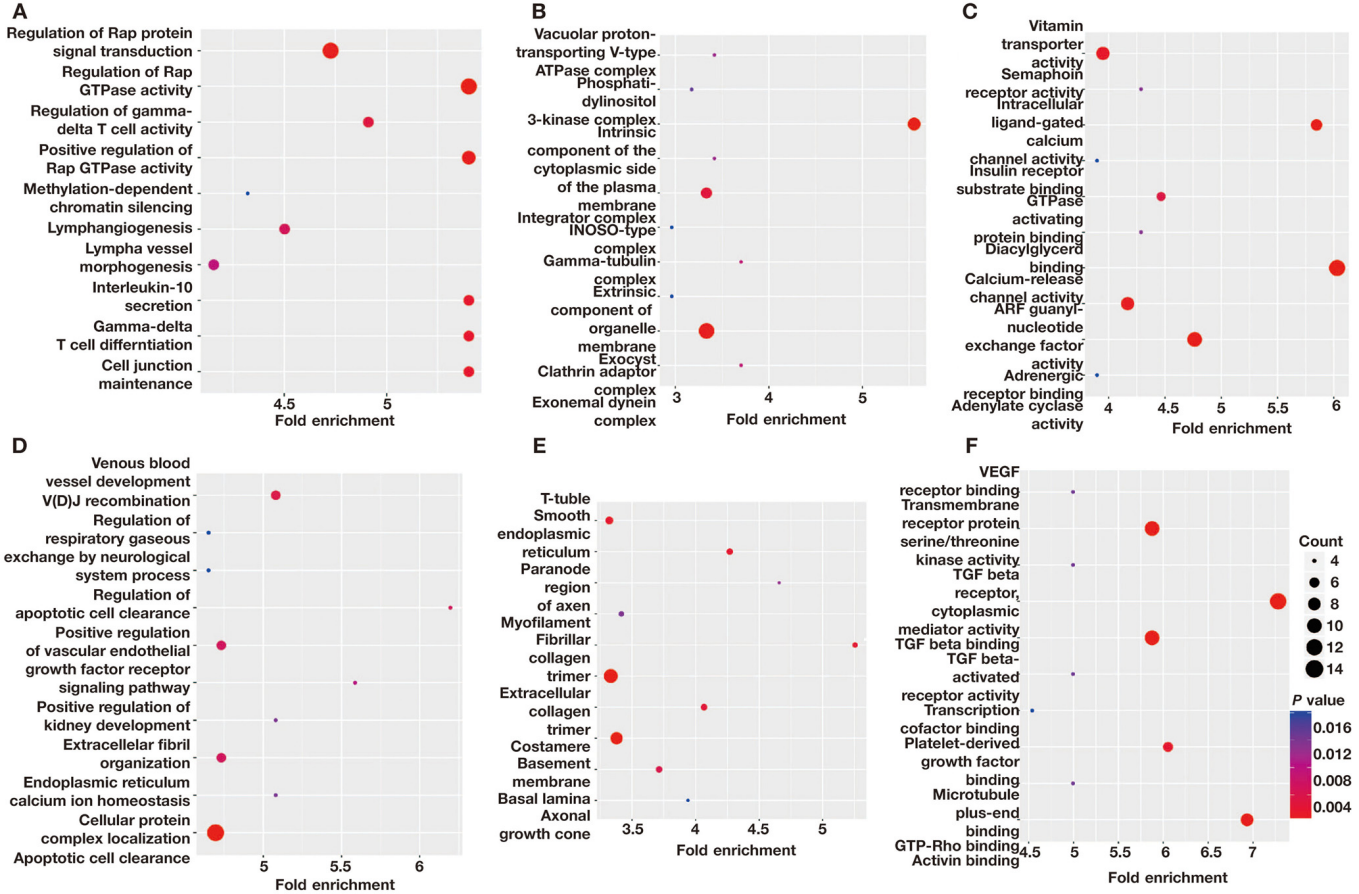
**The GO analysis of DMGs in the LPE vs NP comparison.** (A) The top ten significantly enriched BP terms of hypermethylated DMGs; (B) The top ten significantly enriched CC terms of hypermethylated DMGs; (C) The top ten significantly enriched MF terms of hypermethylated DMGs; (D) The top ten significantly enriched BP terms of hypomethylated DMGs; (E) The top ten significantly enriched CC terms of hypomethylated DMGs; (F) The top ten significantly enriched MF terms of hypomethylated DMGs. DMGs: Differentially methylated mRNAs; EPE: Early-onset preeclampsia; NP: Normal pregnancy; GO: Gene ontology; LPE: Late-onset preeclampsia; BP: Biological process; CC: Cellular component; MF: Molecular function.

**Figure S3.**
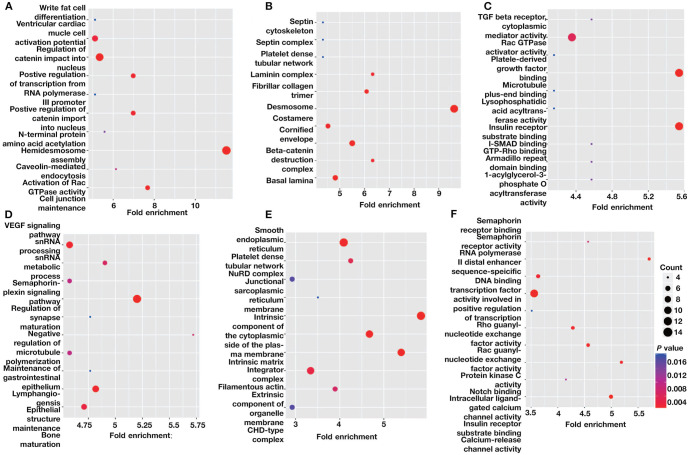
**The GO analysis of DMGs in the EPE vs LPE comparison.** (A) The top ten significantly enriched BP terms of hypermethylated DMGs; (B) The top ten significantly enriched CC terms of hypermethylated DMGs; (C) The top ten significantly enriched MF terms of hypermethylated DMGs; (D) The top 10 significantly enriched BP terms of hypomethylated DMGs; (E) The top ten significantly enriched CC terms of hypomethylated DMGs; (F) The top 10 significantly enriched MF terms of hypomethylated DMGs. DMGs: Differentially methylated mRNAs; EPE: Early-onset preeclampsia; LPE: Late-onset preeclampsia; GO: Gene ontology; LPE: Late-onset preeclampsia; CC: Cellular component; MF: Molecular function.

**Figure S4.**
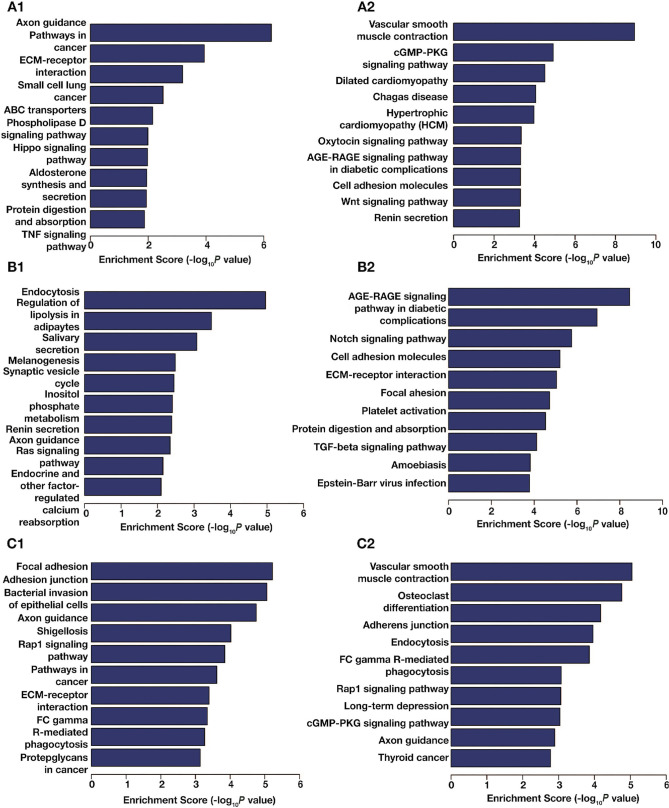
**The KEGG pathway analysis of DMGs in pairwise comparison.** The top ten significantly enriched pathways of hypermethylated (A1) and hypomethylated (A2) DMGs in EPE vs NP. The top ten significantly enriched pathways of hypermethylated (B1) and hypomethylated (B2) DMGs in in LPE vs NP. The top ten significantly enriched pathways of hypermethylated (C1) and hypomethylated (C2) DMGs in EPE vs LPE. The data shown are the negative log_10_ (*P* value) within each category. DMGs: Differentially methylated mRNAs; EPE: Early-onset preeclampsia; NP: Normal pregnancy; LPE: Late-onset preeclampsia; KEGG: Kyoto Encyclopedia of Genes and Genomes.

A comparison of EPE vs NP, LPE vs NP, and EPE vs LPE resulted in 3110, 2801, and 2818 DMGs, respectively. Table 1 presents the top 20 DMGs identified in the pairwise comparisons. To determine the biological significance of m^1^A modifications in preeclamptic decidua, GO and KEGG pathway analyses were conducted for the DMGs (Figures S1–S4). The GO analysis was divided into three functional groups: biological process (BP), cellular component (CC), and molecular function (MF).

**Figure 2.**
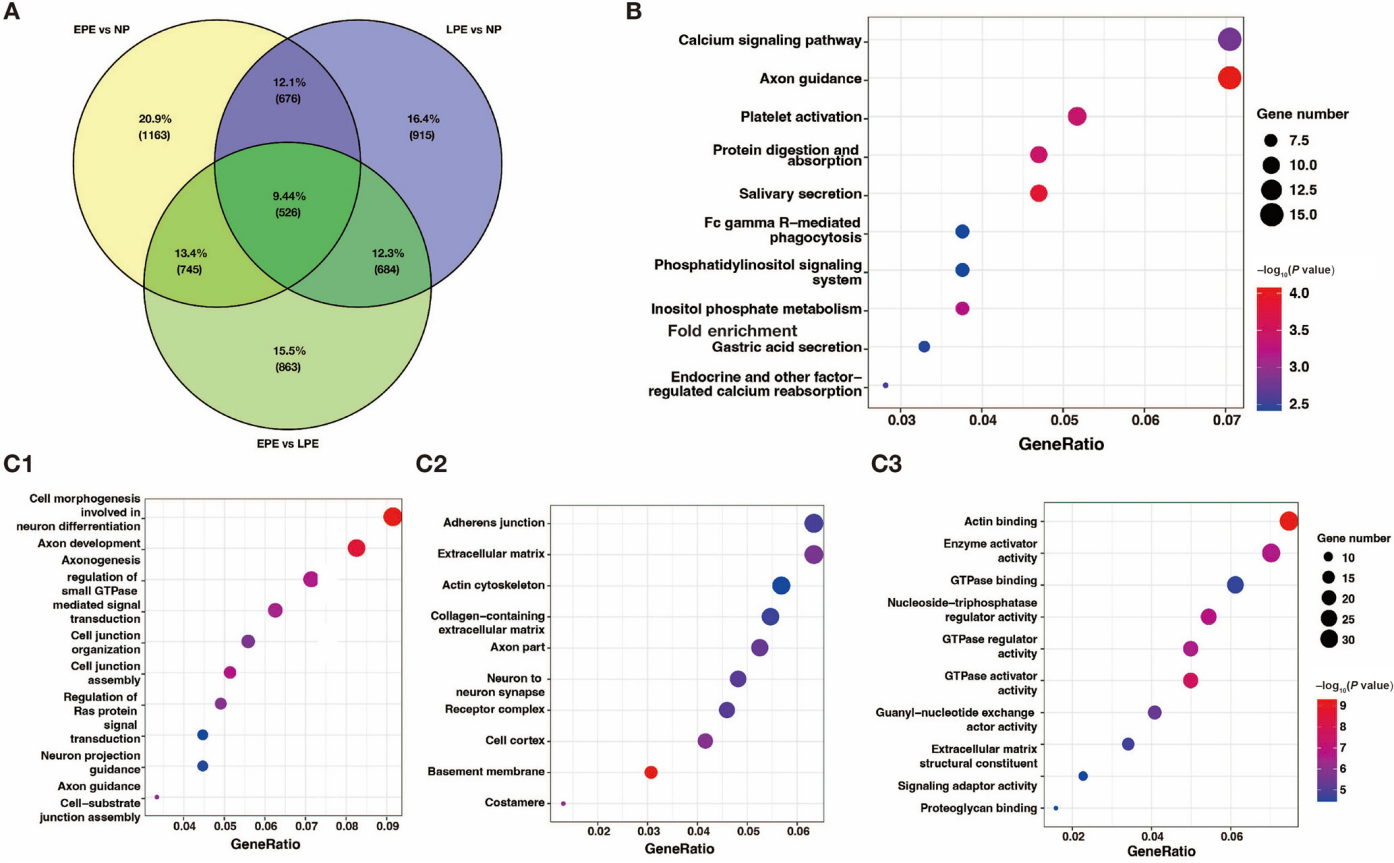
**GO and pathway analysis of key preeclampsia-related DMGs.** (A) Venn diagram depicts the overlapping regions; (B) The top ten significant pathways; (C) A bar plot was generated for the top ten GO terms with the most significant *P* values for biological processes (C1), cellular component (C2), and molecular function (C3). The data shown are the negative log_10_ (*P* value) within each category. EPE: Early-onset preeclampsia; LPE: Late-onset preeclampsia; NP: Normal pregnancy; GO: Gene ontology.

To account for the bias caused by non-matched gestation age in the PE cases and NP controls, a Venn diagram was utilized to visualize the overlaps between the pairwise comparisons [17]. A total of 526 overlapping DMGs were identified as the key PE-related DMGs (Figure 2A). Additionally, KEGG pathway and GO analyses were performed to further elucidate the influence of these key PE-related DMGs. Figure 2B displays the top ten significantly enriched KEGG pathways (*P* < 0.01), including calcium signaling pathway, phosphatidylinositol signaling system, axon guidance, protein digestion and absorption, and others.

**Table 1:** Top 20 differentially methylated m^1^A peaks in pairwise comparisons

**Comparison**	**Chromosome**	**Peak start**	**Peak end**	**Gene name**	**m^1^A methylation**	**Fold change**	***P* value**
EPE vs NP	6	32485153	32485320	*HLA-DRB*	hyper	540.1	0.0000
	3	50197007	50197167	*SEMA3F*	hypo	281.2	0.0000
	4	183245098	183245260	*TENM3*	hyper	76.84	0.0000
	19	49560377	49561120	*CGB7*	hyper	231.5	0.0000
	12	104171621	104171859	*NT5DC3*	hypo	232.6	0.0000
	6	10400680	10400800	*TFAP2A*	hyper	50.44	0.0000
	19	15064946	15065141	*SLC1A6*	hypo	229.3	0.0000
	6	87797830	87797925	*CGA*	hyper	83.41	0.0000
	10	47169786	47169877	*ANXA8*	hyper	31.07	0.0000
	11	64323401	64323840	*SLC22A11*	hyper	42.56	0.0000
	7	29928921	29929023	*WIPF3*	hypo	19.15	0.0000
	6	31686701	31686920	*LY6G6C*	hypo	96.38	0.0000
	11	44954481	44954820	*TP53I11*	hypo	32.12	0.0000
	11	133790161	133791020	*IGSF9B*	hypo	204.4	0.0000
	9	16964	17166	*WASH1*	hyper	15.72	0.0000
	19	50713615	50713960	*MYH14*	hyper	20.14	0.0000
	11	119998121	119998277	*TRIM29*	hyper	62.24	0.0000
	4	75230859	75231092	*EREG*	hyper	213	0.0000
	19	52034835	52035110	*SIGLEC6*	hyper	67.58	0.0000
	7	6661381	6662240	*ZNF853*	hypo	11.16	0.0000
LPE vs NP	21	44339161	44339401	*ERVH48-1*	hyper	407.1	0.0000
	6	87804730	87804865	*CGA*	hyper	283.5	0.0000
	19	2809601	2810488	*THOP1*	hyper	51.03	0.0000
	14	101200941	101201180	*DLK1*	hyper	29.01	0.0000
	19	2794761	2794910	*THOP1*	hyper	15.96	0.0000
	1	186649370	186649559	*PTGS2*	hyper	19.23	0.0000
	19	43773519	43773715	*PSG9*	hyper	12.00	0.0000
	20	25011394	25011420	*ACSS1*	hyper	30.39	0.0000
	15	23261762	23262019	*GOLGA8I*	hyper	11.02	0.0000
	2	202146601	202146638	*CASP8*	hypo	197.4	0.0000
	1	40095898	40095980	*HEYL*	hyper	12.48	0.0000
	19	43766011	43766290	*PSG9*	hyper	579.9	0.0000
	1	36931643	36931960	*CSF3R*	hyper	8.06	0.0000
	X	49597127	49597253	*PAGE4*	hyper	221.5	0.0000
	4	1368881	1369067	*UVSSA*	hyper	7.78	0.0000
	16	89986241	89986740	*MC1R*	hyper	7.73	0.0000
	X	132730393	132730627	*GPC3*	hyper	8.78	0.0000
	7	6661381	6662240	*ZNF853*	hypo	11.53	0.0000
	4	89744141	89744512	*FAM13A*	hyper	11.90	0.0000
	1	204199540	204199714	*PLEKHA6*	hyper	13.14	0.0000
EPE vs LPE	19	43708624	43708766	*PSG4*	hypo	379.00	0.0000
	19	49560377	49561120	*CGB7*	hyper	212.20	0.0000
	19	17936961	17937580	*JAK3*	hypo	20.42	0.0000
	19	15064946	15065141	*SLC1A6*	hyper	210.10	0.0000
	10	47169786	47169877	*ANXA8*	hyper	36.46	0.0000
	19	18901370	18901422	*COMP*	hyper	34.74	0.0000
	19	17462381	17462860	*PLVAP*	hypo	9.63	0.0000
	3	8794761	8794910	*OXTR*	hypo	10.92	0.0000
	14	106053890	106054211	*DKFZp686O16217*	hypo	10.12	0.0000
	15	99672881	99673140	*SYNM*	hypo	7.61	0.0000
	17	74524361	74524693	*CYGB*	hyper	197.70	0.0000
	12	8988161	8988262	*A2ML1*	hyper	193.00	0.0000
	10	47167981	47168360	*ANXA8*	hyper	11.46	0.0000
	12	8988850	8988935	*A2ML1*	hyper	173.10	0.0000
	19	8429341	8429523	*ANGPTL4*	hyper	34.16	0.0000
	2	239148301	239148665	*HES6*	hypo	18.68	0.0000
	3	8808951	8809180	*OXTR*	hypo	7.73	0.0000
	10	119000583	119000731	*SLC18A2*	hyper	14.81	0.0000
	15	63346647	63346740	*TPM1*	hyper	6.63	0.0000
	8	143694781	143695060	*ARC*	hyper	6.93	0.0000

EPE: Early-onset preeclampsia; NP: Normal pregnancy; LPE: Late-onset preeclampsia.

The enriched GO terms, arranged according to BP, CC, and MF, are shown in Figure 2C1–2C3 (*P* < 0.001). The enriched BPs mainly include cell morphogenesis involved in neuron differentiation, regulation of small GTPase-mediated signal transduction, and regulation of Ras protein signal transduction, among others. In terms of CC annotation classification, the key PE-related DMGs are mainly associated with adherens junctions, extracellular matrix, and actin cytoskeleton, among others. Regarding MF, the key PE-related DMGs are mainly localized in actin binding, GTPase activator activity, and signaling adaptor activity, among others.

### Consensus clustering and GSVA of m^1^A regulators

**Figure 3.**
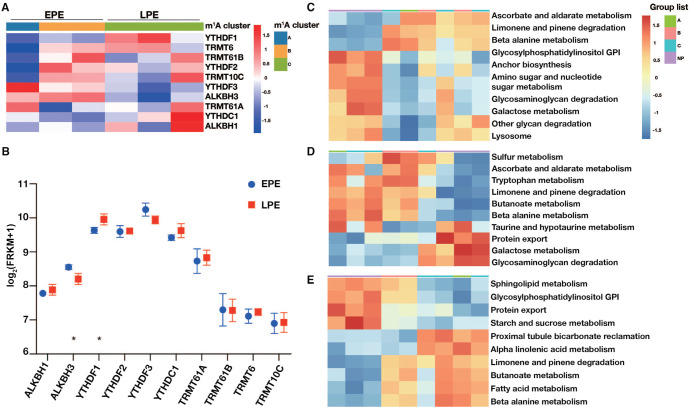
**The consensus clustering and GSVA of m^1^A regulators in preeclamptic decidua.** (A) Heatmap of consensus clustering of 10 m^1^A regulators in PE decidua; (B) The expression levels of 10 m^1^A methylation regulators between EPE and LPE (* *P* < 0.05); (C) Heatmap of GSVA for cluster A in PE and NP decidua; (D) Heatmap of GSVA for cluster B in PE and NP decidua; (E) Heatmap of GSVA for cluster C in PE and NP decidua. GSVA: Gene set variation analysis; PE: Preeclampsia; EPE: Early-onset preeclampsia; LPE: Late-onset preeclampsia; NP: Normal pregnancy.

Based on the expression levels of m^1^A regulators, consensus clustering analysis was performed. *K* ═ 3 was determined to be the optimal selection for dividing PE patients into three clusters: cluster A, cluster B, and cluster C (Figure 3A). Figure 3B shows the expression of 10 m^1^A methylation regulators between EPE and LPE decidua. Compared to EPE, the expression of ALKBH3 was significantly lower in LPE decidua, while the expression of YTHDF1 was significantly higher.

**Figure 4.**
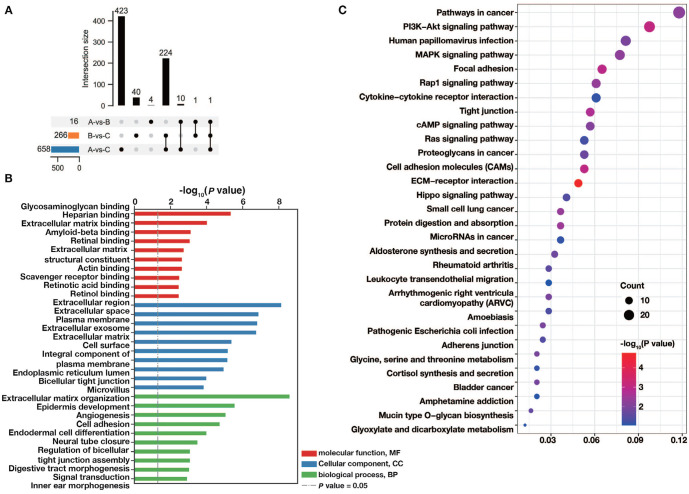
**The functional annotation of m^1^A-related DEGs.** (A) Identification of m^1^A-related DEGs. The functional annotation of m^1^A-related DEGs using GO enrichment analysis (B) and KEGG enrichment analysis (C). DEG: Differentially expressed mRNAs; GO: Gene ontology; KEGG: Kyoto Encyclopedia of Genes and Genomes.

Subsequently, GSVA enrichment analysis of the three m^1^A modification patterns was conducted to investigate the associated pathways and biological significance. Figure 3C shows that m^1^A cluster A is significantly enriched in amino sugar and nucleotide sugar metabolism, glycosaminoglycan degradation, and galactose metabolism, among others. m^1^A cluster B is notably enriched in sulfur metabolism, ascorbate and aldarate metabolism, and tryptophan metabolism, among others (Figure 3D). Meanwhile, m^1^A cluster C is significantly enriched in proximal tubule bicarbonate reclamation, alpha linolenic acid metabolism, limonene and pinene degradation, butanoate metabolism, fatty acid metabolism, and beta alanine metabolism, among others (Figure 3E).

### Functional annotation for m^1^A-related DEGs

A total of 940 m^1^A-related DEGs were identified among three m^1^A modification patterns (Figure 4A). According to the GO analysis, the m^1^A-related DEGs are notably enriched in glycosaminoglycan binding, heparan binding, extracellular matrix binding, and others (MF); extracellular region, extracellular space, plasma membrane, and others (CC); and extracellular matrix organization, epidermis development, angiogenesis, and others (BP) (Figure 4B). Additionally, based on the KEGG analysis, the m^1^A-related DEGs are significantly enriched in the PI3K-Akt signaling pathway, MAPK signaling pathway, Rap1 signaling pathway, cAMP signaling pathway, Ras signaling pathway, and others (Figure 4C).

### Conjoint analysis of DMGs and DEGs

The conjoint analysis of DMGs and DEGs in pairwise comparisons was divided into four categories based on their methylation and expression levels: hypermethylated-upregulated, hypomethylated-downregulated, hypermethylated-downregulated, and hypomethylated-upregulated. The GO analysis of these four categorized DMGs and DEGs is displayed in Table 2.

The comparison between EPE and NP revealed that 356 genes expressed in EPE decidua showed synchronization between m^1^A methylation and transcription levels, while 45 genes showed non-synchronization. The differentially hypermethylated mRNAs were significantly involved in downregulated expressed pathways such as base excision repair and Notch signaling pathway. The upregulated expressed pathways included ECM-receptor interaction, PI3K-Akt signaling pathway, focal adhesion, protein digestion and absorption, and amoebiasis. On the other hand, the differentially hypomethylated mRNAs were significantly involved in downregulated expressed pathways, such as hypertrophic cardiomyopathy, dilated cardiomyopathy, focal adhesion, vascular smooth muscle contraction, and ECM-receptor interaction. The upregulated expressed pathways included oxytocin signaling pathway, lipid and atherosclerosis, nucleocytoplasmic transport, relaxin signaling pathway, and estrogen signaling pathway (Table 3).

The comparison between LPE and NP revealed that 144 genes expressed in LPE decidua showed synchronization between m^1^A methylation and transcription levels, while 62 genes showed non-synchronization. The differentially hypermethylated mRNAs were found to be significantly involved in non-homologous end-joining (downregulated expression), autophagy-animal, Fc gamma R-mediated phagocytosis, longevity regulating pathway-multiple species, synaptic vesicle cycle, and EGFR tyrosine kinase inhibitor resistance (upregulated expression). On the other hand, the differentially hypomethylated mRNAs were significantly involved in ABC transporters, synaptic vesicle cycle, TGF-β signaling pathway, and protein digestion and absorption (downregulated expression). Additionally, focal adhesion, ECM-receptor interaction, MAPK signaling pathway, AGE-RAGE signaling pathway in diabetic complications, and amoebiasis were identified as being upregulated (Table 3).

The comparison between EPE and LPE revealed that 218 genes expressed in EPE decidua were synchronized between m^1^A methylation and transcription levels, while 50 genes were unsynchronized. The differentially hypermethylated mRNAs were significantly involved in downregulated expression of aldosterone-regulated sodium reabsorption, endocrine and other factor-regulated calcium reabsorption, and steroid hormone biosynthesis. For the upregulated expression, they were involved in ECM-receptor interaction, protein digestion and absorption, human papillomavirus infection, focal adhesion, and small cell lung cancer. Regarding the differentially hypomethylated mRNAs, they were significantly involved in downregulated expression of vascular smooth muscle contraction, oxytocin signaling pathway, axon guidance, transcriptional misregulation in cancer, and hypertrophic cardiomyopathy. For the upregulated expression, they were involved in non-homologous end-joining, arginine biosynthesis, terpenoid backbone biosynthesis, and thyroid cancer (Table 3).

**Table 2:** The top five significant GO terms of DMGs and DEGs in conjoint analysis

**Pairwise comparisons**	**DMGs methylation-DEGs expression pattern**	**GO terms**	**Genes involved**	***P* value**
EPE vs NP	hyper-down	BP		
		Notch signaling pathway	*FOXC1, MAML3, TSPAN14*	0.0003
		Protein localization to plasma membrane	*LRP6, DPP6, TSPAN14*	0.0006
		Positive regulation of Notch signaling pathway	*MAML3, TSPAN14*	0.0007
		Demethylation	*KDM1B, TDG*	0.0009
		Protein localization to cell periphery	*LRP6, DPP6, TSPAN14*	0.0009
		CC		
		Tetraspanin-enriched microdomain	*TSPAN14*	0.0069
		Wnt signalosome	*LRP6*	0.0083
		Centriolar satellite	*SDCCAG8*	0.0207
		Nuclear heterochromatin	*FOXC1*	0.0227
		Plasma membrane protein complex	*LRP6, DPP6*	0.0381
		MF		
		Transcription coregulator activity	*FOXC1, MAML3, TDG*	0.0056
		Mismatched DNA binding	*TDG*	0.0076
		Chloride ion binding	*TDG*	0.0076
		Low-density lipoprotein particle receptor activity	*LRP6*	0.0092
		DNA N-glycosylase activity	*TDG*	0.0099
	hyper-up	BP		
		Hemidesmosome assembly	*COL17A1, ITGB4, LAMC2, LAMB3, KRT14, KRT5, LAMA3*	0.0000
		Epidermis development	*KRT24, COL17A1, KRT6A, ERRFI1, DSC3, LAMC2, DSP, FLNB, LAMB3, INHBA, KRT19, KRT5, KRT7, LAMA3, AGPAT2, PTCH2, KRT8, SFN*	0.0000
		Skin development	*COMP, COL5A1, KRT24, ITGB4, KRT6A, ERRFI1, DSC3, DSP, FLNB, INHBA, KRT19, KRT14, KRT5, KRT7, PTCH2, KRT8, SFN*	0.0000
		Cell-substrate junction	*COL17A1, FN1, ITGB4, LAMC2, LAMB3, KRT14, KRT5, PTK2B, LAMA3*	0.0000
		Cornification	*KRT24, KRT6A, DSC3, DSP, KRT19, KRT14, KRT5, KRT7, KRT8*	0.0000
		CC		
		Extracellular matrix	*COMP, COL5A1, MMP15, COL17A1, FN1, COL27A1, LAMC2, FBN2, SERPINE2, LAMB3, SERPINE1, LAMA3, PZP, DST, GPC1, COL28A1, TFPI2, ADAMTSL4*	0.0000
		Collagen-containing extracellular matrix	*COMP, COL5A1, COL17A1, FN1, LAMC2, FBN2, SERPIN2, LAMB3, SERPINE1, LAMA3, PZP, DST, GPC1, COL28A1, ADAMTSL4*	0.0000
		Basement membrane	*COL5A1, COL17A1, FN1, LAMC2, LAMB3, LAMA3, DST, COL28A1*	0.0000
		Extracellular matrix component	*COL5A1, COL27A1, LAMC2, FBN2, LAMB3, LAMA3*	0.0000
		Cell cortex	*SCIN, LAMC2, FLNB, SH3BP1, KRT19, PTK2B, DST, FNBP1L, KCNC3*	0.0000
		MF		
		Extracellular matrix structural constituent	*COMP, COL5A1, COL17A1, FN1, COL27A1, LAMC2, FBN2, LAMB3, LAMA3, COL28A1, TFPI2*	0.0000
		Serine-type endopeptidase inhibitor activity	*SPINT2, SERPINE2, SERPINE1, PZP, COL28A1*	0.0000
		Structural constituent of cytoskeleton	*KRT6A, DSP, KRT19, KRT14, KRT5*	0.0001
		Extracellular matrix structural constituent conferring tensile strength	*COL5A1, COL17A1, COL27A1, COL28A1*	0.0001
		Cell adhesion molecule binding	*COMP, COL5A1, FN1, IGF2, DSP, FLNB, DST, FNBP1L, COBLL1, SFN*	0.0004
	hypo-down	BP		
		Muscle system process	*TPM2, DES, LMOD1, CNN1, CALD1, ACTG2, IGF1, MYL9*	0.0000
		Muscle contraction	*TPM2, DES, LMOD1, CNN1, CALD1, ACTG2, MYL9*	0.0000
		Negative regulation of smooth muscle cell apoptotic process	*LRP6, IGF1*	0.0004
		Cellular response to ethanol	*KCNMB1, TP53INP1*	0.0004
		Positive regulation of glycolytic process	*ZBTB20, IGF1*	0.0008
		CC		
		Myofibril	*TPM2, DES, LMOD1, SYNPO2, CALD1, MYL9*	0.0000
		Contractile fiber	*TPM2, DES, LMOD1, SYNPO2, CALD1, MYL9*	0.0000
		Sarcomere	*TPM2, DES, LMOD1, SYNPO2, MYL9*	0.0000
		Contractile fiber part	*TPM2, DES, LMOD1, SYNPO2, MYL9*	0.0000
		Actin cytoskeleton	*TPM2, LMOD1, SYNPO2, CALD1, ACTG2, MYL9*	0.0003
		MF		
		Integrin binding	*VWF, SPP1, IGF1*	0.0017
		Structural constituent of muscle	*TPM2, MYL9*	0.0026
		Extracellular matrix structural constituent	*MMRN2, SBSPON, VWF*	0.0045
		Actin binding	*TPM2, LMOD1, CNN1, CALD1*	0.0069
		Myosin binding	*CALD1, MYL9*	0.0072
	hypo-up	BP		
		Nucleotide biosynthetic process	*NUP188, TECR, NOS3, AAAS, KMO*	0.0000
		Nucleotide phosphate biosynthetic process	*NUP188, TECR, NOS3, AAAS, KMO*	0.0000
		Tissue homeostasis	*LDB2, MUC6, NOS3, LRRK1*	0.0001
		Carboxylic acid biosynthetic process	*NUP188, PLOD2, TECR, AAAS, KMO*	0.0001
		Organic acid biosynthetic process	*NUP188, PLOD2, TECR, AAAS, KMO*	0.0002
		CC		
		Cytoplasmic microtubule	*PAFAH1B1, SNPH*	0.0024
		Host	*NUP188, AAAS*	0.0030
		Host cell	*NUP188, AAAS*	0.0030
		Other organism	*NUP188, AAAS*	0.0035
		Other organism cell	*NUP188, AAAS*	0.0035
		MF		
		Oxidoreductase activity, acting on paired donors, with incorporation or reduction of molecular oxygen	*PLOD2, NOS3, KMO*	0.0006
		Organic anion transmembrane transporter activity	*SLCO2B1, SLC25A29, SLC6A20*	0.0006
		Oxidoreductase activity, acting on paired donors, with incorporation or reduction of molecular oxygen, NAD(P)H as one donor, and incorporation of one atom of oxygen	*NOS3, KMO*	0.0007
		Amino acid transmembrane transporter activity	*SLC25A29, SLC6A20*	0.0021
		Flavin adenine dinucleotide binding	*NOS3, KMO*	0.0031
LPE vs NP	hyper-down	BP		
		Somatic hypermutation of immunoglobulin gene	*POLM*	0.0086
		Somatic diversification of immune receptor via somatic mutation	*POLM*	0.0093
		Sperm axoneme assembly	*IQCG*	0.0093
		Chaperone cofactor-dependent protein refolding	*DNAJC7*	0.0101
		snRNA processing	*INTS8*	0.0117
		CC		
		Integrator complex	*TNTS8*	0.0120
		MF		
		Heat shock protein binding	*DNAJC7, IQCG*	0.0030
		DNA-directed DNA polymerase activity	*POLM*	0.0160
		GTP-dependent protein binding	*RAPGEF5*	0.0167
		Chemokine binding	*PLP2*	0.0175
		DNA polymerase activity	*POLM*	0.0242
	hyper-up	BP		
		Adherens junction assembly	*DLG5, PHLDB2, MMP14, KDR*	0.0003
		Regulation of cell junction assembly	*PHLDB2, RAPGEF2, MMP14, KDR*	0.0003
		Phagosome maturation	*SYT7, UNC13B, ATP6V0A1*	0.0004
		Positive regulation of vasculogenesis	*RAPGEF2, KDR*	0.0005
		Positive regulation of regulated secretory pathway	*SYT7, UNC13B, GAB2*	0.0005
		CC		
		Extracellular matrix	*FBN2, TFPI2, SPOCK2, SERPINB9, MUC4, GPC1, MMP14, PKM*	0.0001
		Golgi lumen	*FURIN, MUC4, GPC1, MMP14*	0.0003
		Early phagosome	*SYT7, UNC13B*	0.0007
		Phagocytic vesicle	*SYT7, UNC13B, ATP6V0A1, UVRAG*	0.0008
		Endocytic vesicle	*SYT7, RAPGEF2, UNC13B, ATP6V0A1, UVRAG*	0.0023
		MF		
		Growth factor binding	*IGFBP1, FURIN, GPC1, IGF1R, KDR*	0.0000
		Cadherin binding	*PHLDB2, TAGLN2, TRIM25, KDR, ASAP1, PKM, CAPZB*	0.0000
		Cell adhesion molecule binding	*PHLDB2, TAGLN2, TRIM25, MMP14, KDR, ASAP1, PKM, CAPZB*	0.0002
		Phospholipid binding	*SYT7, SCIN, ZCCHC2, RAPGEF2, UNC13B, GAB2, ASAP1*	0.0003
		Phosphatidylinositol bisphosphate binding	*SYT7, SCIN, GAB2, ASAP1*	0.0004
	hypo-down	BP		
		Glutamate secretion	*PPFIA3, SLC1A1*	0.0003
		Acidic amino acid transport	*PPFIA3, SLC1A1*	0.0006
		Import across plasma membrane	*ABCC9, SLC1A1*	0.0007
		Dicarboxylic acid transport	*PPFIA3, SLC1A1*	0.0014
		Acid secretion	*PPFIA3, SLC1A1*	0.0021
		CC		
		Cell cortex region	*PPFIA3*	0.0181
		Photoreceptor connecting cilium	*LCA5*	0.0210
		Presynaptic active zone	*PPFIA3*	0.0348
		Ciliary transition zone	*LCA5*	0.0354
		Potassium channel complex	*ABCC9*	0.0461
		MF		
		Transforming growth factor beta receptor, cytoplasmic mediator activity	*SMAD9*	0.0065
		L-glutamate transmembrane transporter activity	*SLC1A1*	0.0065
		Pseudouridine synthase activity	*RPUSD3*	0.0065
		Acidic amino acid transmembrane transporter activity	*SLC1A1*	0.0065
		Glutamate binding	*SLC1A1*	0.0065
	hypo-up	BP		
		Extracellular matrix organization	*ADAM19, NFKB2, COL1A2, FN1*	0.0020
		Post-translation protein modification	*LTBP1, FN1, FBXL22, FSTL3*	0.0026
		Extracellular structure organization	*ADAM19, NFKB2, COL1A2, FN1*	0.0033
		Protein-DNA complex assembly	*RSF1, UBTF, RAD23B*	0.0046
		Cell junction assembly	*GPBAR1, FN1, PEAK1*	0.0049
		CC		
		Collagen-containing extracellular matrix	*ADAM19, LTBP1, COL1A2, FN1, PSAP*	0.0003
		Extracellular matrix	*ADAM19, LTBP1, COL1A2, FN1, PSAP*	0.0006
		Endoplasmic reticulum lumen	*LTBP1, COL1A2, FN1, FSTL3*	0.0011
		Extracellular matrix component	*LTBP1, COL1A2*	0.0022
		SW1/SNF superfamily-type complex	*RSF1, CHD4*	0.0046
		MF		
		Protease binding	*COL1A2, FN1, PSAP*	0.0011
		Growth factor binding	*FLT1, LTBP1, COL1A2*	0.0011
		Extracellular matrix structural constituent	*LTBP1, COL1A2, FN1*	0.0024
		Transmembrane receptor protein kinase activity	*FLT1, LTBP1*	0.0055
		Ceramide binding	*PSAP*	0.0170
EPE vs LPE	hyper-down	BP		
		Platelet degranulation	*CFD, SCCPDH*	0.0044
		Cytoskeletal anchoring at plasma membrane	*TLN2*	0.0078
		Regulation of morphogenesis of an epithelium	*NTN4, AP2A2*	0.0085
		Bleb assembly	*PMP22*	0.0086
		Positive regulation of systemic arterial blood pressure	*HSD11B2*	0.0093
		CC		
		Platelet alpha granule lumen	*CFD, SCCPDH*	0.0013
		Platelet alpha granule	*CFD, SCCPDH*	0.0024
		AP-2 adaptor complex	*AP2A2*	0.0088
		Ficolin-1-rich granule	*CFD, AP2A2*	0.0094
		Clathrin coat of endocytic vesicle	*AP2A2*	0.0096
		MF		
		Phospholipid-translocating ATPase activity	*ATP10D*	0.0088
		Carbohydrate: cation symporter activity	*SLC45A4*	0.0097
		Solute: proton symporter activity	*SLC45A4*	0.0097
		Magnesium ion binding	*ATP10D, WEE1*	0.0143
		Laminin binding	*NTN4*	0.0227
	hyper-up	BP		
		Hemidesmosome assembly	*COL17A1, KRT14, LAMB3, LAMA3*	0.0000
		Cell-substrate junction assembly	*COL17A1, KRT14, LAMB3, S100A10, LAMA3, CLASP2*	0.0000
		Epidermis development	*COL17A1, KRT14, LAMB3, KRT24, RPSS8, KRT6A, KRT8, PTCH2, LAMA3*	0.0000
		Cornification	*KRT14, KRT24, PRSS8, KRT6A, KRT8*	0.0000
		Cell junction assembly	*COL17A1, KRT14, LAMB3, S100A10, LAMA3, CLASP2*	0.0000
		CC		
		Collagen-containing extracellular matrix	*COL17A1, LAMB3, PZP, COMP, S100A10, LAMA3, COL28A1*	0.0000
		Basement membrane	*COL17A1, LAMB3, LAMA3, COL28A1*	0.0000
		Extracellular matrix	*COL17A1, LAMB3, PZP, COMP, S100A10, LAMA3, COL28A1*	0.0000
		Costamere	*AHNAK2, KRT8*	0.0006
		Intermediate filament	*KRT14, KRT24, KRT6A, KRT8*	0.0007
		MF		
		Extracellular matrix structural constituent	*COL17A1, LAMB3, COMP, LAMA3, COL28A1*	0.0000
		Extracellular matrix structural constituent conferring tensile strength	*COL17A1, COL28A1*	0.0034
		Collagen binding	*COMP, GP6*	0.0074
		Guanyl-nucleotide exchange factor activity	*DOCK5, PSD4, KALRN*	0.0074
		Proton transmembrane transporter activity	*SLC9A3, ATP6V1E2*	0.0082
	hypo-down	BP		
		Muscle system process	*MEF2C, CNN1, MYH11, ACTG2, MYL9, DES, OXTR, CALD1, TPM2, DDX39B*	0.0000
		Muscle contraction	*CNN1, MYH11, ACTG2, MYL9, DES, OXTR, CALD1, TPM2*	0.0000
		Regulation of development growth	*MEF2C, ULK2, MAP18, RBP4, DDX39B, HLX*	0.0002
		Regulation of organ growth	*MEF2C, RBP4, DDX39B, HLX*	0.0002
		Regulation of chemokine-mediated signaling pathway	*SLIT3, ROBO1*	0.0003
		CC		
		Contractile fiber	*MYH11, MYL9, SYNPO2, DES, CALD1, TPM2*	0.0000
		Actin cytoskeleton	*MYH11, ACTG2, PDLIM7, MYL9, SYNPO2, CALD1, TPM2*	0.0000
		Contractile fiber part	*MYH11, MYL9, SYNPO2, DES, TPM2*	0.0001
		Myofibril	*MYL9, SYNPO2, DES, CALD1, TPM2*	0.0001
		Stress fiber	*PDLIM7, MYL9, SYNPO2*	0.0004
		MF		
		Structural constituent of muscle	*MYH11, MYL9, TPM2*	0.0001
		RNA polymerase II distal enhancer sequence-specific DNA binding	*MEF2C, SPI1, FLI1*	0.0014
		Actin binding	*CNN1, MYH11, CALD1, TNS1, TPM2*	0.0021
		Enhancer sequence-specific DNA binding	*MEF2C, SPI1, FLI1*	0.0025
		Enhancer binding	*MEF2C, SPI1, FLI1*	0.0035
	hypo-up	BP		
		Sulfur compound catabolic process	*CSPG4, FMOD*	0.0012
		Glycosaminoglycan catabolic process	*CSPG4, FMOD*	0.0019
		Aminoglycan catabolic process	*CSPG4, FMOD*	0.0020
		Somatic diversification of immune receptors	*TCF7, POLM*	0.0023
		Cell redox homeostasis	*NOS3, SCO2*	0.0024
		CC		
		Lysosomal lumen	*CSPG4, FMOD*	0.0041
		Golgi lumen	*CSPG4, FMOD*	0.0048
		Vacuolar lumen	*CSPG4, FMOD*	0.0129
		Integrator complex	*INTS4*	0.0152
		Lamellipodium membrane	*CSPG4*	0.0222
		MF		
		FMN binding	*NOS3*	0.0137
		MAP kinase activity	*MAP3K13*	0.0210
		DNA-directed DNA polymerase activity	*POLM*	0.0221
		Protein disulfide oxidoreductase activity	*SCO2*	0.0221
		Extracellular matrix structural constituent conferring compression resistance	*FMOD*	0.0221

DMGs: Differentially methylated mRNAs; DEGs: Differentially expressed mRNAs; EPE: Early-onset preeclampsia; LPE: Late-onset preeclampsia; NP: Normal pregnancy; BP: Biological processes; CC: Cellular component; MF: Molecular function.

### Validation of external datasets for the differential expression of key m^1^A-related DEGs and m^1^A modulators

**Figure S5.**
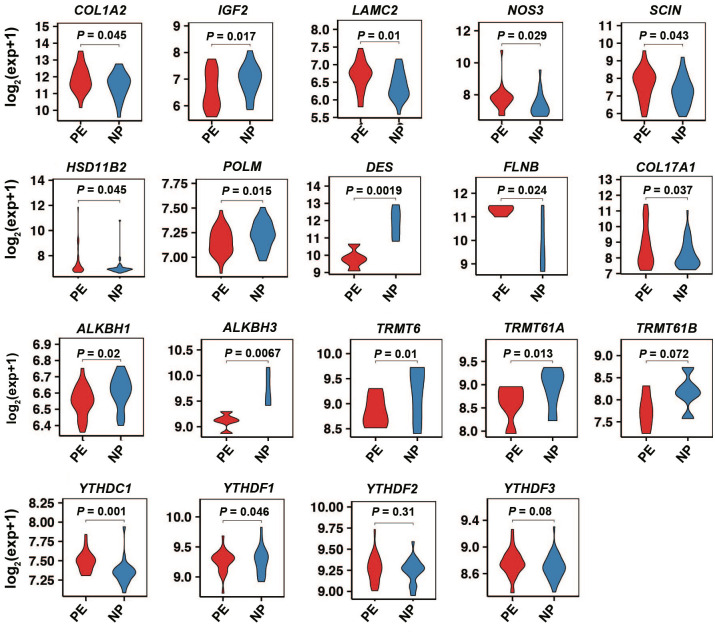
**The external validation of the mRNA expression of several key m^1^A-related DEGs and m^1^A modulators.** The significant differences in the expression of *COL1A2*, *IGF2*, *LAMC2*, *NOS3*, *SCIN*, *HSD11B2, POLM*, *DES*, *FLNB*, *COL17A1*, *ALKBH1*, *ALKBH3*, *TRMT61B*, *YTHDC1,* and *YTHDF1* between preeclamptic decidua and normal pregnant decidua. m^1^A: *N*^1^-methyladenosine; DEG: Differentially expressed mRNAs.

For external validation of the key m^1^A-related DEGs, we consulted the GEO dataset, GSE60438, which comprised gene expression data from 60 PE patients and 65 normal samples. We observed significant differences in the mRNA expression of *COL1A2, IGF2, LAMC2, NOS3*, *SCIN*, *HSD11B2, POLM, DES*, *FLNB*, and *COL17A1* between PE decidua and NP decidua (Figure S5). These findings further underscore the reliability of our study. Additionally, we also observed significant differences in the mRNA expression of *ALKBH1*, *ALKBH3*, *YTHDC1*, and *YTHDF1* among 10 m^1^A modulators between preeclamptic decidua and normal pregnant decidua (Figure S5).

## Discussion

Accumulated evidence reveals that disorders related to m^1^A are widely associated with the pathogenesis of many diseases. To our knowledge, this study is the first to investigate the m^1^A landscape in preeclamptic decidua using MeRIP-seq. The results of this study demonstrate distinct m^1^A modification profiles in the decidua of PE compared to NP. A total of 3110, 2801, and 2818 DMGs were identified when comparing EPE and NP, LPE and NP, and EPE and LPE, respectively. Further, a combined analysis of MeRIP-seq and RNA-seq data was employed to identify genes associated with the differential expression of methylated m^1^A peaks and differentially expressed mRNA levels. In addition, we categorized three m^1^A modification patterns based on 10 m^1^A regulators. Functional annotation analysis of key PE-related DMGs and m^1^A-related DEGs revealed their involvement in decidualization and pathways related to vascular dysfunction. These findings highlight the strong relationship between m^1^A methylation and the decidualization process in preeclamptic decidua.

Until now, there has been no consensus on the etiology of PE. Recent research has focused primarily on the early stage of decidualization, which allows for implantation. Human epithelial stroma cells (ESCs) derived from nonpregnant women with a history of sPE showed a lack of differentiation, as indicated by the absence of structural changes and secretory markers. Further, conditioned medium from sPE decidual cells was unable to induce cytotrophoblast invasion [5]. Decidualization is a transient phase of the endometrium that makes it receptive to embryonic signaling and promotes implantation. This process occurs before the embryo reaches the uterine cavity and is driven by ovarian hormones such as estrogen and progesterone, as well as an increase in local cAMP production. The cAMP signaling pathway, activated by progesterone, plays a crucial role in sensitizing HESCs and stimulating the transcriptional activity of the progesterone receptor [18]. Decidualization in HESCs is initiated through the rapid non-genomic ERK/MAPK pathway [19]. In this study, we identified DMGs and DEGs that are closely associated with essential biological pathways involved in the decidualization process.

The Notch signaling mechanism is a highly conserved developmental pathway that is essential for implantation and placentation. In primates, HESCs respond to chorionic gonadotropin and progesterone by activating the Notch receptor 1 (NOTCH1) pathway. NOTCH1 induces the expression of α-smooth muscle actin and positively regulates cytoskeletal remodeling and the initial changes typical of the decidualization process [20]. Dysfunction in both the loss and gain of Notch signaling leads to impairment of endometrial function [21]. The PI3K/serine/threonine protein kinase (Akt) pathway is known to affect the synthesis of decidual markers [22], and inhibition of this pathway could be involved in the decrease in motility of HESCs during decidualization [23]. TGF-β is composed of three isoforms, including TGF-β1, TGF-β2, and TGF-β3. Emerging data demonstrate an important interplay between the TGF-β signaling pathway and decidualization [24]. TGF-β1 plays an essential role in regulating fetal-maternal immune tolerance during pregnancy [25]. Elevated levels of decidual TGF-β1 suppress the activation of specific subsets of decidual natural killer cells, which in turn contributes to the uteroplacental pathology associated with the onset of PE [26]. This study revealed that the differentially hypomethylated mRNAs in LPE decidua were significantly involved in the TGF-β signaling pathway, showing downregulated expression compared to NP decidua (Table 3). However, the role of the TGF-β signaling pathway in the pathological development of PE is still highly contradictory and requires further elucidation [27].

**Table 3:** The top five significant pathways of DMGs and DEGs in conjoint analysis

**Pairwise comparisons**	**DMGs methylation-DEGs expression pattern**	**Pathway terms**	**Genes involved**	***P* value**
EPE vs NP	hyper-down	Base excision repair	*TDG*	0.0201
		Notch signaling pathway	*MAML3*	0.0359
	hyper-up	ECM-receptor interaction	*COMP, FN1, ITGB4, LAMC2, LAMB3, LAMA3, GP6*	0.0000
		PI3K-Akt signaling pathway	*COMP, FN1, ITGB4, IGF2, LAMC2, LAMB3, DDIT4, FLT1, RPTOR, LAMA3, NOS3*	0.0000
		Focal adhesion	*COMP, FN1, ITGB4, LAMC2, FLNB, LAMB3, FLT1, LAMA3*	0.0000
		Protein digestion and absorption	*COL5A1, COL17A1, COL27A1, SLC9A3, MME, COL28A1*	0.0000
		Amoebiasis	*FN1, IL1R2, LAMC2, LAMB3, LAMA3*	0.0006
	hypo-down	Hypertrophic cardiomyopathy	*ITGA7, TPM2, DES, IGF1*	0.0000
		Dilated cardiomyopathy	*ITGA7, TPM2, DES, IGF1*	0.0001
		Focal adhesion	*ITGA7, VWF, SPP1, IGF1, MYL9*	0.0002
		Vascular smooth muscle contraction	*KCNMB1, CALD1, ACTG2, MYL9*	0.0004
		ECM-receptor interaction	*ITGA7, VWF, SPP1*	0.0016
	hypo-up	Oxytocin signaling pathway	*NFATC3, GNAO1, NOS3*	0.0021
		Lipid and atherosclerosis	*NFATC3, NOS3, IRF7*	0.0053
		Nucleocytoplasmic transport	*NUP188, AAAS*	0.0143
		Relaxin signaling pathway	*GNAO1, NOS3*	0.0201
		Estrogen signaling pathway	*GNAO1, NOS3*	0.0227
LPE vs NP	hyper-down	Non-homologous end-joining	*POLM*	0.0048
	hyper-up	Autophagy-animal	*RPTOR, DAPK1, IGF1R, UVRAG*	0.0021
		Fc gamma R-mediated phagocytosis	*SCIN, GAB2, ASAP1*	0.0063
		Longevity regulating pathway-multiple species	*RPTOR, IGF1R*	0.0244
		Synaptic vesicle cycle	*UNC13B, P6V0A1*	0.0373
		EGFR tyrosine kinase inhibitor resistance	*IGF1R, KDR*	0.0382
	hypo-down	ABC transporters	*ABCC9*	0.0219
		Synaptic vesicle cycle	*SLC1A1*	0.0378
		TGF-beta signaling pathway	*SMAD9*	0.0454
		Protein digestion and absorption	*SLC1A1*	0.0496
	hypo-up	Focal adhesion	*FLT1, COL1A2, FN1*	0.0035
		ECM-receptor interaction	*COL1A2, FN1*	0.0083
		MAPK signaling pathway	*FLT1, NFKB2, MKNK2*	0.0102
		AGE-RAGE signaling pathway in diabetic complications	*COL1A2, FN1*	0.0107
		Amoebiasis	*COL1A2, FN1*	0.0111
EPE vs LPE	hyper-down	Aldosterone-regulated sodium reabsorption	*HSD11B2*	0.0270
		Endocrine and other factor-regulated calcium reabsorption	*AP2A2*	0.0384
		Steroid hormone biosynthesis	*HSD11B2*	0.0441
	hyper-up	ECM-receptor interaction	*LAMB3, COMP, LAMA3, GP6*	0.0000
		Protein digestion and absorption	*COL17A1, SLC9A3, COL28A1*	0.0022
		Human papillomavirus infection	*LAMB3, COMP, LAMA3, ATP6V1E2*	0.0093
		Focal adhesion	*LAMB3, COMP, LAMA3*	0.0142
		Small cell lung cancer	*LAMB3, LAMA3*	0.0231
	hypo-down	Vascular smooth muscle contraction	*MYH11, ACTG2, MYL9, CALD1*	0.0006
		Oxytocin signaling pathway	*MEF2C, MYL9, OXTR*	0.0100
		Axon guidance	*MYL9, SLIT3, ROBO1*	0.0157
		Transcriptional misregulation in cancer	*MEF2C, SPI1, FLI1*	0.0184
		Hypertrophic cardiomyopathy	*DES, TPM2*	0.0285
	hypo-up	Non-homologous end-joining	*POLM*	0.0159
		Arginine biosynthesis	*NOS3*	0.0267
		Terpenoid backbone biosynthesis	*RCE1*	0.0267
		Thyroid cancer	*TCF7*	0.0445

DMGs: Differentially methylated mRNAs; DEGs: Differentially expressed mRNAs; EPE: Early-onset preeclampsia; LPE: Late-onset preeclampsia; NP: Normal pregnancy.

Further, maternal vascular dysfunction is another feature of PE that plays a role in its pathogenesis and can persist into the postpartum period [28]. Potential abnormalities include impaired placentation, incomplete spiral artery remodeling, and endothelial damage, which consequently lead to placental ischemia [29]. When placental ischemia occurs, bioactive factors are released into the maternal circulation and cause an imbalance between antiangiogenic soluble fms-like tyrosine kinase-1 and soluble endoglin, and proangiogenic vascular endothelial growth factor (VEGF), placental growth factor (PGF), and TGF-β. These bioactive factors target vascular smooth muscle and impact vascular contraction mechanisms, leading to increased vasoconstriction in PE [30]. This study also revealed that differentially hypomethylated mRNAs in the EPE decidua were significantly involved in downregulated expression of vascular smooth muscle contraction pathways compared to both LPE and NP groups (Table 3).

Additionally, the uterine vascular adaptation to pregnancy is modulated by ovarian hormones, including estrogen and relaxin, which affect both the endothelium and the vascular smooth muscle [31, 32]. This study discovered the differentially hypomethylated mRNAs in EPE decidua were significantly involved in the upregulated expression of pathways, including relaxin signaling pathway and estrogen signaling pathway (Table 3). Relaxin, a peptide hormone secreted by the corpus luteum and circulating during pregnancy, exhibits local expression in decidua cells along with its major receptor, relaxin/insulin-like family peptide receptor 1 (RXFP1) [33]. Several clinical studies have documented a significant change in the levels of estradiol and relaxin during PE [34, 35]. Plenty of evidence demonstrates that estrogen-induced uterine vasodilation in pregnancy is through the estrogen-VEGF-NO pathway [32, 36]. Also, VEGF and PGF are emerging as essential mediators in relaxin-induced vasodilation [37, 38]. Therefore, the disturbance of estrogen signaling pathway will decrease uterine vascular resistance and induce uterine hemodynamic changes which are associated with PE.

Additionally, we have noted that the number of unique m^1^A methylated mRNAs in NPs is consistently higher than in both EPE and LPE (Figure 1). This suggests a broader range of m^1^A modifications in NPs compared to those with PE, potentially due to altered regulatory mechanisms or stress responses in preeclamptic conditions. Some researchers have utilized m^1^A modulators for cancer detection, prognostication, clinical staging, and as targets for drug-based disease treatment [39]. Moreover, in our study, we have observed significant differences in the mRNA expression of *ALKBH1*, *ALKBH3*, *YTHDC1*, and *YTHDF1* among 10 m^1^A modulators between preeclamptic decidua and normal pregnant decidua through external dataset validation (Figure S5). Therefore, it is imperative to undertake further research and development efforts to refine m^1^A modulation patterns in decidualization, enabling their versatile application.

There are clear limitations in this study, including the relatively small sample size for a heterogeneous disease like PE. Additionally, the patients in our study are primarily of Asian ethnicity, which may limit the generalizability of our findings to other populations, such as Caucasian or Sub-Saharan African groups. Furthermore, gestational age differences could contribute to the observed modifications. Although we included pairwise comparisons between normal pregnancies and late-onset preeclamptic cases, which had more comparable gestational ages than early-onset preeclamptic cases, the differences in gestational age between normal and preeclamptic decidua could influence the results. This makes it challenging to distinguish whether the observed modifications are due to the disease state or differences in gestational age and highlights the need for further studies to control for this variable more rigorously.

## Conclusion

In summary, the present study has revealed that PE decidua exhibits significantly different mRNA m^1^A modification patterns and gene expression profiles. These differences impact various signaling pathways related to decidualization and vascular dysfunction. The distinct m^1^A modification patterns observed provide valuable insight into how the process of decidualization and vascular function contribute to the development of PE. These m^1^A modification regulators could potentially be used as biomarkers or therapeutic targets for PE, necessitating further investigation.

## Supplemental data

## Data Availability

The data that support the findings of this study are available from the corresponding author upon reasonable request.
